# Disseminated Tuberculosis Presenting As Scrofuloderma and Tuberculous Meningitis With Ischemic Stroke in the Absence of a Pulmonary Disease

**DOI:** 10.7759/cureus.108498

**Published:** 2026-05-08

**Authors:** Alejandro Rivera, Horacio Muñoz, Tiffany A Dunaway

**Affiliations:** 1 Internal Medicine, Hospital Universitario Dr. José Eleuterio González, Monterrey, MEX

**Keywords:** cutaneous tuberculosis, extrapulmonary tuberculosis, ischemic stroke, scrofuloderma, tuberculous meningitis

## Abstract

Extrapulmonary tuberculosis represents a significant proportion of tuberculosis cases, and scrofuloderma is an uncommon cutaneous manifestation caused by contiguous spread from underlying structures, such as lymph nodes, bone, or joints. Its association with central nervous system involvement, particularly tuberculous meningitis, is rare and carries a high risk of morbidity and mortality if not promptly recognized. We report the case of a 69-year-old man who initially presented with painful, indurated supraclavicular and anterior thoracic skin lesions that progressed to ulceration with seropurulent discharge, clinically consistent with scrofuloderma. This was followed by intermittent low-grade fever and subsequent subacute neurological deterioration, including altered mental status, headache, somnolence, ataxia, and disorientation. On admission, he was febrile and found to have severe hyponatremia, a frequent metabolic complication of tuberculous meningitis. Cerebrospinal fluid analysis revealed lymphocytic pleocytosis, markedly low glucose, and elevated protein and lactate, consistent with tuberculous meningitis. Brain magnetic resonance imaging demonstrated right insular leptomeningeal enhancement and a subacute ischemic infarction in the right corona radiata, suggestive of tuberculous vasculitis. Histopathological examination and GeneXpert testing (Cepheid; Sunnyvale, CA, USA) of a skin biopsy confirmed infection with *Mycobacterium tuberculosis *(MTB), establishing the diagnosis in the absence of pulmonary involvement. Antituberculous therapy and adjunctive corticosteroids were promptly initiated, with favorable clinical evolution and progressive neurological improvement. This case highlights scrofuloderma as a potential initial manifestation of disseminated tuberculosis and underscores the importance of maintaining a high index of suspicion for tuberculous meningitis in patients presenting with chronic ulcerative skin lesions and neurological deterioration, even without a pulmonary disease, as early diagnosis and treatment are critical to improving outcomes.

## Introduction

Extrapulmonary tuberculosis accounts for approximately 10-20% of all tuberculosis cases and may involve almost any organ system [1]. Cutaneous tuberculosis is an uncommon manifestation, representing less than 2% of cases, and includes a spectrum of clinical entities [2]. Scrofuloderma is the most frequent form of cutaneous tuberculosis and results from contiguous spread from underlying infected structures, most commonly lymph nodes, bone, or joints, leading to subcutaneous nodules that may progress to ulceration and sinus tract formation [2,3].

Tuberculous meningitis is one of the most severe forms of extrapulmonary tuberculosis and is associated with high morbidity and mortality [4]. Its subacute presentation often delays diagnosis, and complications, such as cerebral vasculitis, ischemic stroke, and metabolic disturbances, may contribute to neurological deterioration [4,5]. The occurrence of scrofuloderma as the initial clinical manifestation of disseminated tuberculosis with subsequent central nervous system involvement is rare. We report a case of scrofuloderma preceding the diagnosis of tuberculous meningitis complicated by leptomeningitis and ischemic stroke in the absence of a pulmonary disease, highlighting the diagnostic value of cutaneous lesions and the importance of early recognition of central nervous system tuberculosis.

## Case presentation

**Figure 1:**
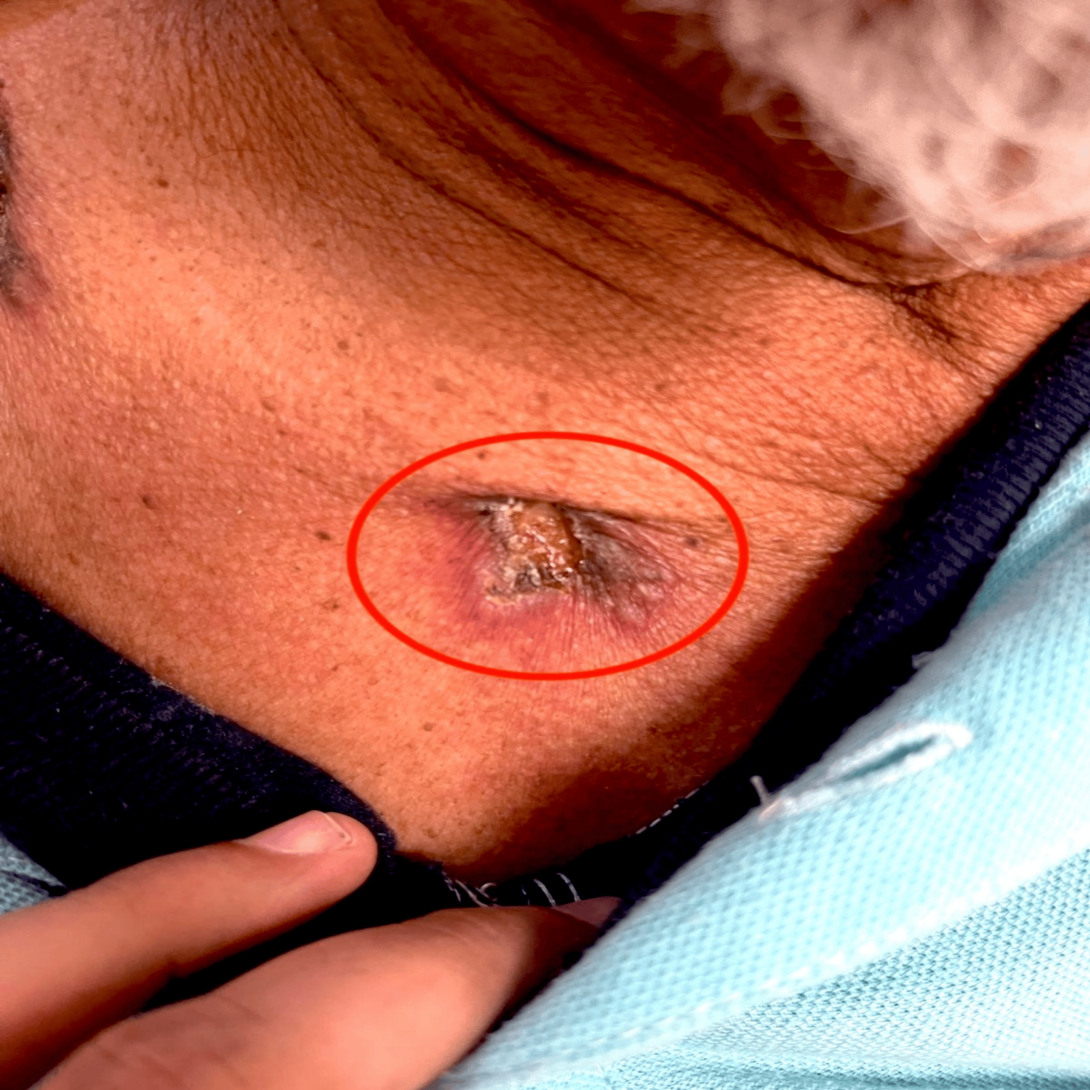
Ulcerated plaque in the right supraclavicular region, measuring approximately 5-6 cm and characterized by a central necrotic crust with seropurulent exudate, surrounded by induration and violaceous erythema, consistent with scrofuloderma.

A 69-year-old man with a history of former tobacco and alcohol use and no known recent tuberculosis exposure presented with progressive cutaneous and neurological symptoms. Four months before admission, he developed a painful, indurated mass measuring approximately 5-6 cm in the right supraclavicular region (Figure 1). Over the following weeks, similar lesions appeared in the right supraclavicular area and anterior thoracic wall. These lesions gradually evolved into ulcerated plaques with seropurulent, nonfetid drainage and tenderness on palpation. During this period, he reported intermittent low-grade fever but denied respiratory symptoms, such as cough, dyspnea, hemoptysis, or weight loss. The chronic, progressive nature of the lesions was clinically suggestive of scrofuloderma. He had no history of HIV infection, diabetes, or immunosuppressive conditions.

One week before presentation, the patient developed progressive neurological symptoms, including somnolence, hyporexia, diaphoresis, and holocranial headache, followed by altered mental status, disorientation, incoherent speech, gait instability, and ataxia. Upon arrival at the emergency department, he was febrile (38.5°C) and hemodynamically stable. On physical examination, he appeared ill and exhibited decreased responsiveness, with a Glasgow Coma Scale score of 13 (E3V4M6). Neurological examination revealed no focal motor deficits, with preserved muscle strength in all extremities and symmetric deep tendon reflexes. Pupils were equal and reactive to light, and no cranial nerve deficits were identified. Signs of meningeal irritation were present, including neck stiffness and a positive Kernig’s sign. Initial laboratory evaluation revealed severe hyponatremia (118 mEq/L), which partially improved after hypertonic saline administration. Given the subacute neurological deterioration, a diagnostic lumbar puncture was performed.

**Figure 2:**
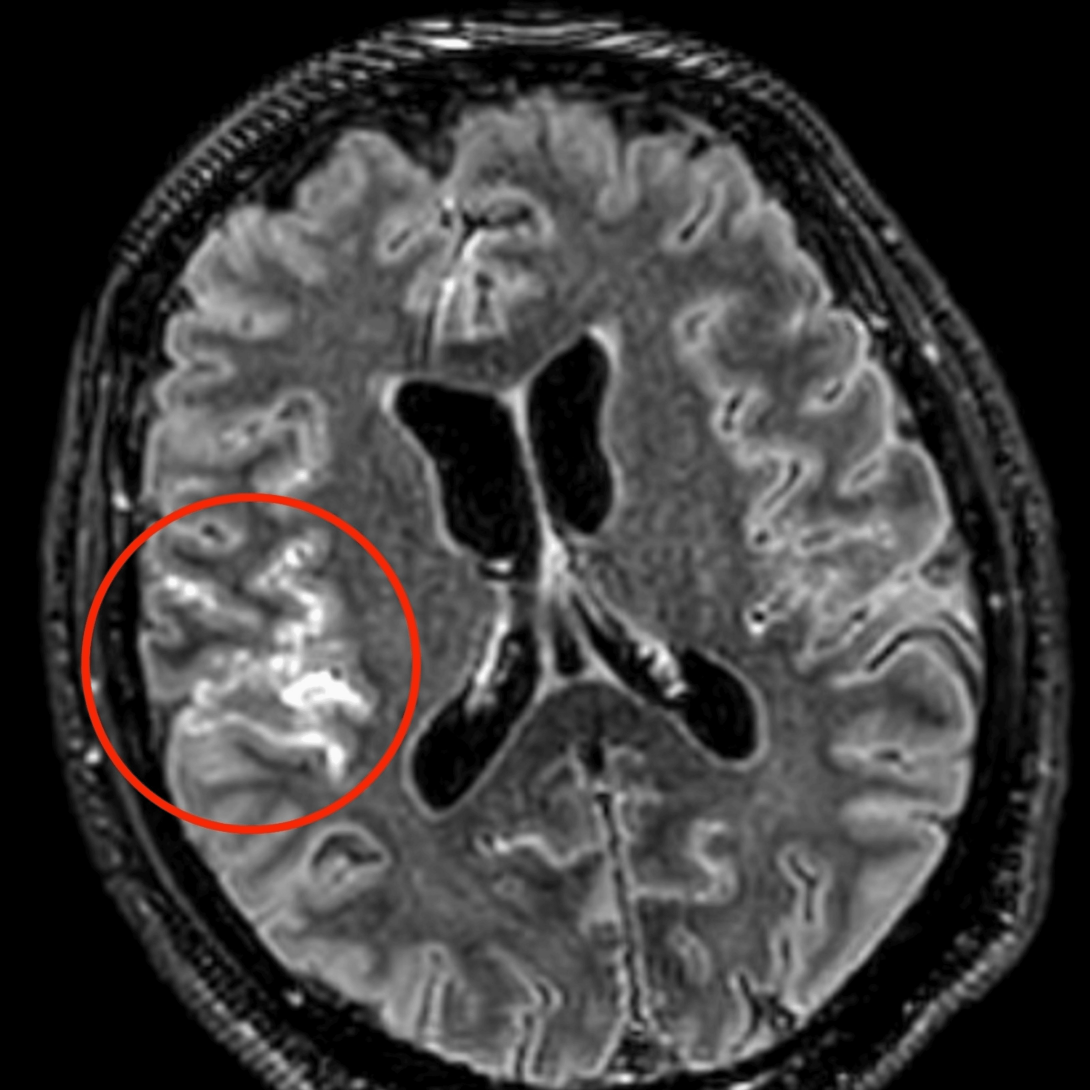
Axial post-contrast FLAIR image demonstrating focal leptomeningeal enhancement in the right posterior frontal region, adjacent to the insular cortex, consistent with leptomeningitis in the setting of tuberculous meningitis (red circle indicates the area of leptomeningeal enhancement). FLAIR: fluid-attenuated inversion recovery

**Figure 3:**
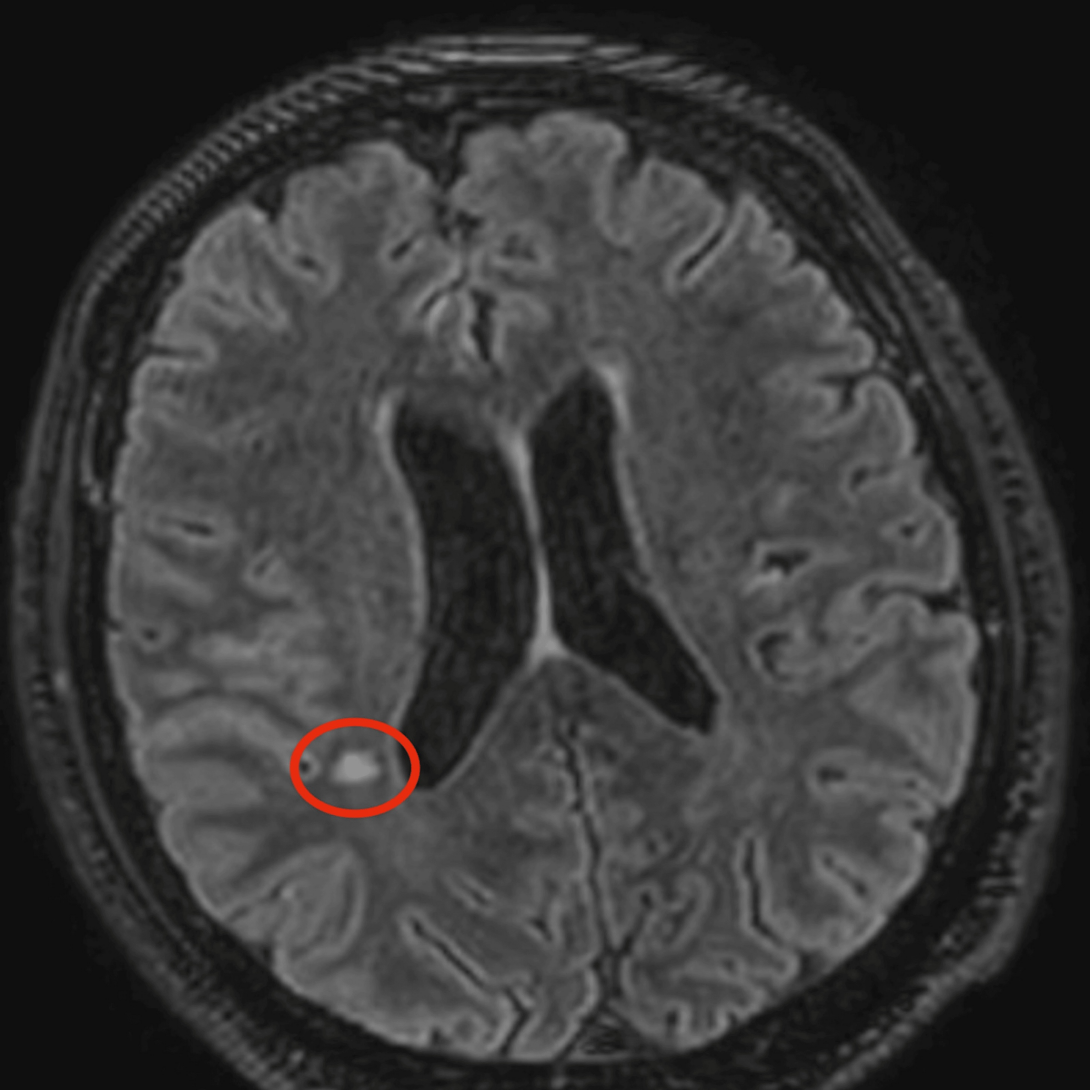
Axial post-contrast FLAIR MRI demonstrating focal leptomeningeal enhancement in the right posterior frontal region, adjacent to the insular cortex, with an adjacent cortical-subcortical hyperintense lesion consistent with subacute ischemic infarction (red circle highlights the cortical-subcortical hyperintense lesion). FLAIR: fluid-attenuated inversion recovery; MRI: magnetic resonance imaging

**Figure 4:**
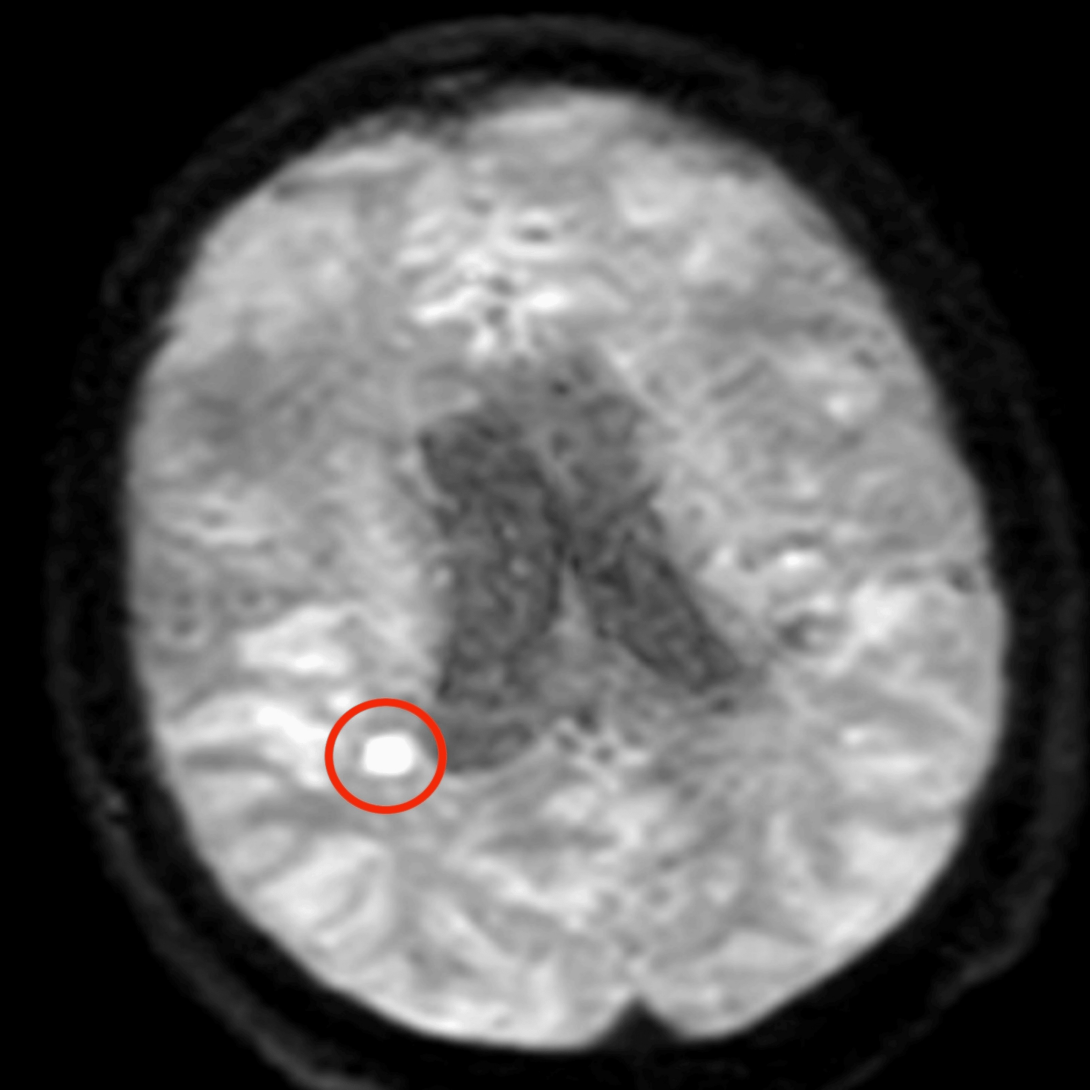
Axial diffusion-weighted (DW) MRI demonstrating a focal hyperintense lesion in the right posterior frontal cortical-subcortical region, adjacent to the insular cortex, consistent with acute ischemic infarction (red circle indicates the area of restricted diffusion). MRI: magnetic resonance imaging

Cerebrospinal fluid analysis demonstrated turbid fluid with 270 cells/mm³ (90% lymphocytes), glucose of 17 mg/dL, protein of 153 mg/dL, and lactate of 3.7 mmol/L (Table 1), findings highly suggestive of tuberculous meningitis. Gram stain of the cerebrospinal fluid was negative. No nucleic acid amplification test or additional molecular diagnostic studies were performed on the cerebrospinal fluid due to financial constraints. Cerebrospinal fluid cultures for *Mycobacterium tuberculosis* (MTB) were obtained and were negative after eight weeks of incubation. At the time of the latest follow-up, final culture results were still unavailable. Viral meningitis panel and rapid testing for HIV, hepatitis B virus, and hepatitis C virus were negative. Brain magnetic resonance imaging revealed leptomeningeal enhancement predominantly involving the right insular region (Figure 2), as well as a subacute ischemic infarction in the right corona radiata (Figures 3, 4). Diffuse meningeal enhancement was also noted, raising concern for tuberculous vasculitis.

**Table 1:** Cerebrospinal fluid (CSF) analysis at admission. CSF analysis revealed lymphocytic pleocytosis, hypoglycorrhachia, hyperproteinorrachia, and low chloride levels, findings consistent with tuberculous meningitis.

Parameter	Result	Reference range
Appearance	Slightly turbid	Clear
White blood cell count	270 leukocytes/mm³	0-5 leukocytes/mm³
Polymorphonuclear cells	10%	Not specified
Lymphocytes	90%	Not specified
Erythrocytes	Scant, non-hemolyzed	None
Gram stain	Negative	Negative
Glucose	17 mg/dL	45-80 mg/dL
Protein	153 mg/dL	15-45 mg/dL
Chloride	103.7 mmol/L	118.1-132.0 mmol/L
pH	8.5	Not specified
Albumin	0.1 g/dL	3.2-5.5 g/dL
Lactate	3.7 mmol/L	0.3-11.0 mmol/L

Given the concomitant cutaneous findings, a skin biopsy of one of the ulcerated lesions was obtained. Histopathological examination revealed granulomatous inflammation composed of epithelioid histiocytes and lymphocytes, with areas of caseous necrosis, findings characteristic of cutaneous tuberculosis. In addition, a nucleic acid amplification test was performed on the skin biopsy specimen using the GeneXpert MTB/RIF assay (Cepheid; Sunnyvale, CA, USA), a real-time polymerase chain reaction-based test that detects MTB complex DNA and evaluates rifampin (RIF) resistance. The assay yielded a positive result for MTB, confirming the diagnosis. Chest imaging showed no radiographic evidence of pulmonary tuberculosis.

The patient was admitted to the internal medicine service and promptly started on antituberculous therapy with a standard four-drug regimen consisting of isoniazid, RIF, pyrazinamide, and ethambutol, along with adjunctive dexamethasone. Multidisciplinary care was provided in conjunction with neurology, infectious diseases, and dermatology services. During hospitalization, he developed hyperactive delirium and persistent electrolyte disturbances, including recurrent hyponatremia attributed to syndrome of inappropriate antidiuretic hormone secretion, which were managed with fluid restriction and electrolyte correction. Transcranial Doppler ultrasonography performed during follow-up showed no evidence of cerebral vasospasm. No additional sites of tuberculosis involvement, including spinal or adrenal disease, were systematically investigated during hospitalization.

Over the course of a two-week hospital stay, the patient demonstrated progressive clinical improvement, with resolution of fever, stabilization of electrolyte abnormalities, and gradual recovery of neurological function. At the time of discharge, no residual focal neurological deficits were identified, and the patient had returned to his baseline mental status. He was discharged on continued antituberculous therapy and a tapering course of corticosteroids, with outpatient follow-up arranged. At follow-up three months after discharge, the patient demonstrated sustained clinical improvement, with favorable evolution of the cutaneous lesions. No clinical images of the lesions were obtained during subsequent evaluations.

## Discussion

Cerebrovascular complications in tuberculous meningitis are primarily mediated by inflammatory vasculitis secondary to the rupture of meningeal Rich foci, leading to vessel wall infiltration, luminal narrowing, and subsequent cerebral infarction [6]. This inflammatory vasculopathy represents one of the most severe consequences of central nervous system tuberculosis and is strongly associated with adverse neurological outcomes. Ischemic stroke has been reported in approximately one-quarter to one-third of patients with tuberculous meningitis and is a major determinant of long-term neurological sequelae. Although tuberculous meningitis is more frequently described in individuals with immunosuppression, it may also occur in immunocompetent patients, in whom delayed recognition is common due to lower clinical suspicion [4,7]. The occurrence of ischemic stroke in this case underscores the aggressive inflammatory nature of meningeal tuberculosis, even in the absence of classic risk factors. Adjunctive corticosteroid therapy plays a critical role in the management of tuberculous meningitis by attenuating the host inflammatory response, reducing meningeal exudates and vasculitis, and lowering the risk of neurological complications, including cerebral infarction. In this context, negative cerebrospinal fluid cultures do not exclude the diagnosis, as microbiological confirmation in tuberculous meningitis is often limited by low bacillary load.

Scrofuloderma represents the most common form of cutaneous tuberculosis and reflects contiguous spread from an underlying tuberculous focus, most often lymph nodes or bone [2,3]. Its presence should prompt systematic evaluation for deeper or disseminated disease. The concomitant occurrence of scrofuloderma and tuberculous meningitis, particularly in the absence of pulmonary involvement, is exceedingly rare and only sporadically reported, limiting precise estimates of incidence. This combination is clinically significant, as early recognition of cutaneous manifestations may facilitate prompt identification of disseminated disease and prevent severe neurological complications. In this patient, chronic ulcerative cutaneous lesions preceded neurological deterioration, highlighting the importance of recognizing cutaneous tuberculosis as a potential early indicator of systemic involvement. Early initiation of standard antituberculous therapy combined with adjunctive corticosteroids (isoniazid, RIF, pyrazinamide, and ethambutol, along with adjunctive dexamethasone) is essential to improve outcomes. Prognosis in such cases is highly dependent on the timing of diagnosis and initiation of therapy, with delayed treatment associated with an increased risk of permanent neurological sequelae and mortality. Differential diagnosis of cutaneous manifestations included chronic bacterial infection, deep fungal infection, cutaneous malignancy, and inflammatory dermatoses; however, progressive course, anatomical distribution, and histopathological findings supported a tuberculous etiology. Microbiological confirmation through GeneXpert testing (Cepheid; Sunnyvale, CA, USA) of a skin biopsy was pivotal in establishing the diagnosis, particularly in the absence of pulmonary findings, reinforcing the diagnostic value of cutaneous tissue sampling in disseminated tuberculosis.

## Conclusions

This case emphasizes the clinical importance of recognizing chronic ulcerative cutaneous lesions, such as scrofuloderma, as potential markers of underlying disseminated tuberculosis rather than isolated dermatologic conditions. Careful assessment of these lesions can prompt early consideration of systemic involvement and guide targeted diagnostic testing, allowing timely confirmation of tuberculosis even in the absence of a pulmonary disease. In this context, cutaneous biopsy represents a valuable and accessible diagnostic tool that may facilitate earlier diagnosis of occult extrapulmonary tuberculosis.

In addition, this report highlights that severe manifestations of tuberculosis, including tuberculous meningitis, complicated by cerebral infarction, can occur in immunocompetent individuals without traditional risk factors. A high index of suspicion is therefore essential when cutaneous and neurological findings coexist, as delayed recognition may result in irreversible neurological injury. Prompt initiation of antituberculous therapy and adjunctive corticosteroids remains fundamental to improving neurological outcomes and reducing long-term morbidity in tuberculous meningitis.

## References

[1] (2026). World Health Organization. Global tuberculosis report 2022. https://www.who.int/publications/i/item/9789240061729.

[2] Frankel A, Penrose C, Emer J (2009). Cutaneous tuberculosis: a practical case report and review for the dermatologist. J Clin Aesthet Dermatol.

[3] Santos JB, Figueiredo AR, Ferraz CE, Oliveira MH, Silva PG, Medeiros VL (2014). Cutaneous tuberculosis: epidemiologic, etiopathogenic and clinical aspects - part I. An Bras Dermatol.

[4] Donovan J, Thwaites GE, Huynh J (2023). Tuberculous meningitis. Lancet.

[5] Chen X, Chen F, Liang C (2023). MRI advances in the imaging diagnosis of tuberculous meningitis: opportunities and innovations. Front Microbiol.

[6] Rock RB, Olin M, Baker CA, Molitor TW, Peterson PK (2008). Central nervous system tuberculosis: pathogenesis and clinical aspects. Clin Microbiol Rev.

[7] Wilkinson RJ, Rohlwink U, Misra UK (2017). Tuberculous meningitis. Nat Rev Neurol.

